# Investigation of Readout RF Pulse Impact on the Chemical Exchange Saturation Transfer Spectrum

**DOI:** 10.1038/srep15062

**Published:** 2015-10-12

**Authors:** Sheng-Min Huang, Meei-Ling Jan, Hsin-Chin Liang, Chia-Hao Chang, Yi-Chun Wu, Shang-Yueh Tsai, Fu-Nien Wang

**Affiliations:** 1 Department of Biomedical Engineering and Environmental Sciences, National Tsing Hua University, Hsinchu 30013, Taiwan; 2 Health Physics Division, Institute of Nuclear Energy Research, Lungtan, Taoyuan 32546, Taiwan; 3 Center for Advanced Molecular Imaging and Translation, Chang Gung Memorial Hospital, Taoyuan 333, Taiwan; 4 Graduate Institute of Applied Physics, National Chengchi University, Taipei 11605, Taiwan; 5 Research Center for Mind, Brain and Learning, National Chengchi University, Taipei 11605, Taiwan

## Abstract

Chemical exchange saturation transfer magnetic resonance imaging (CEST-MRI) is capable of both microenvironment and molecular imaging. The optimization of scanning parameters is important since the CEST effect is sensitive to factors such as saturation power and field homogeneity. The aim of this study was to determine if the CEST effect would be altered by changing the length of readout RF pulses. Both theoretical computer simulation and phantom experiments were performed to examine the influence of readout RF pulses. Our results showed that the length of readout RF pulses has unremarkable impact on the Z-spectrum and CEST effect in both computer simulation and phantom experiment. Moreover, we demonstrated that multiple refocusing RF pulses used in rapid acquisition with relaxation enhancement (RARE) sequence induced no obvious saturation transfer contrast. Therefore, readout RF pulse has negligible effect on CEST Z-spectrum and the optimization of readout RF pulse length can be disregarded in CEST imaging protocol.

CEST has emerged as a novel and promising MRI technique in the last several years. The CEST effect is sensitive to factors such as exchange rates, temperature, solute concentration, and pH values. Furthermore, the exchangeable protons will respond to any change and achieve a new chemical equilibrium condition by exchanging energy. Utilizing the CEST-MRI technique, water proton signals are attenuated by the exchangeable labile protons of contrast agents after radio-frequency (RF) pulse saturation. This characteristic provides a special contrast of microenvironment properties, such as concentration of contrast agent, pH value, and temperature. Thus, the CEST contrast can reflect physiological changes in any tissue and assist in understanding of the mechanism of diseases^1^. Furthermore, the rapid development of CEST contrast agents makes CEST capable of probing biologically and pathologically relevant molecules^2^, including glucose^3^, enzymes^4^, inorganic ions and metabolites^5^,^6^. Medical applications such as the grading of tumors^7^,^8^, characterization of ischemia^9^,^10^,^11^,^12^ and monitoring of drug delivery^13^ were also discovered.

To generate CEST contrast, magnetization preparation pulse is embedded in the pulse sequence. The most commonly used method is a continuous wave (CW) RF irradiation followed by a fast acquisition pulse sequence, such as echo-planar imaging (EPI). The scanning process includes saturation irradiation pulse design, RF pulse length, readout RF pulse type and duration, and other sequence parameters. For small molecule contrast agents like creatine and amide protons, the CEST effect is usually susceptible and thus the optimization of the abovementioned scanning parameters is essential.

Small molecule contrast agents often have chemical shifts with only several ppms from central frequency^14^. For example, amide proton transfer (APT) imaging has been reported to be useful for detecting lactic acidosis during acute ischemia; the chemical shift of the APT mechanism is within 1~4 ppm^15^. Sun *et al.* studied the optimization of saturation irradiation power^16^. For the saturation RF pulse, especially in small molecule contrast agents, a lot of research studies have evaluated options for shortening imaging time and gaining CEST effects. Sun *et al.*^17^ compared pulse-CEST with the continuous wave irradiation method to optimize the CEST effect. They found that the optimal RF power and maximal CEST contrast for both irradiation schemes were approximately equal. Horska and Spencer^18^ correctly accounted for direct irradiation of RF pulse in a saturation exchange experiment. The direct irradiation (spillover effect) would influence the accuracy of the measured Z-spectrum and must be corrected.

To date, many studies have been conducted on the optimization of sequence design and saturation RF power^19^,^20^. However, little literature is available on the effects of readout RF pulses in the image acquisition sequences. As illustrated in Fig. 1, a conventional CEST pulse sequence contains preparation part and readout part, where a spin-echo readout consists of excitation (90°) and refocusing (180°) RF pulses. To the best of our knowledge, the issue of readout RF pulses was only briefly mentioned in Sun’s work^21^. They speculated that readout RF pulse is not long enough to generate CEST contrast. Since the bandwidth of readout RF is related to the pulse length, readout RF pulse length thus may be a factor that influencing CEST effect due to spillover effect. The purpose of this study was thus to investigate if the CEST effect would be altered by readout RF including excitation and refocusing pulses. Both theoretical simulations and phantom experiments were carried out to examine this issue using steady state saturation preparation process.

## Results

### Computer simulation results

Figure 2 shows the simulated Z-spectrum and MTR asymmetry (MTR_asym_) based on single spin-echo imaging with various RF pulse durations. In Fig. 2(a), there is no obvious difference among various RF pulse lengths. Figure 2(b) shows the zoomed-in Z-spectrum between −4.85 ppm and −5 ppm. A trend was found that long RF pulse length would result in a broader Z-spectrum. The signal difference of Z-spectrum between 0.5 ms and 10 ms is around 0.05% at −5 ppm offset. For the MTR_asym_ of creatine, the difference is from 0.01% to 0.07%, as listed in Table 1. However, no specific trend was found along with RF durations.

Figure 3 shows the simulated result from multiple refocusing RF pulses: 1, 2, 4, and 8 refocusing pulses were simulated. Among the four Z-spectra there is no observable difference. If we take a closer look to the Z-spectra, 8 refocusing RF pulses would result in a narrower Z-spectrum. Nevertheless, the signal difference at −5 ppm offset between 1 refocusing RF pulse and 8 refocusing RF pulses is smaller than 0.0025%.

### MRI phantom experiment

Figure 4 shows the phantom image acquired by EPI. ROI were selected for Z-spectrum evaluation and MTR_asym_ calculation. The Z-spectra of background gel, creatine and barbituric acid are displayed in Fig. 5. Figure 5(b) shows the Z-spectra of gel obtained with various RF pulse lengths. Since the variation from noise is around 0.5% in the phantom study, the trend found in simulation results would be covered by noise. Statistical analysis showed that there is no significant difference between each two Z-spectra. (Z-spectra differences were calculated and subjected to two-tails t-test against zero, p = 0.14 ~ 0.62) As listed in Table 1, there is no specific trend along with RF durations for the MTR_asym_ of creatine.

Figure 6 shows the comparison of Z-spectra between EPI and RARE method. The Z-spectrum obtained by EPI was slightly narrower than the Z-spectrum acquired by RARE (Z-spectra differences were calculated and subjected to two-tails t-test against zero, p = 0.0362). The 50% width (S_z_/S_0_ = 0.5) of the Z-spectrum was 819 Hz in EPI result and was 834 Hz in RARE result.

## Discussions

Small molecule CEST contrast agents usually have a chemical shift around 1 ~ 5 ppm. Taking APT contrast as an example, the chemical shift is around 3.5 ppm, and the corresponding frequency offset is 700 Hz at 4.7 T main field. A RF pulse length of 1 ms has a corresponding excitation bandwidth of 2740 Hz, where the frequency offset of APT would be covered in excitation process. Therefore, we hypothesized it might be possible that during excitation, the RF pulse would excite exchangeable protons on small chemical shift contrast agents, resulting in an additional chemical exchange effect and increasing the total CEST ratio.

Since the bandwidth in long RF pulse is smaller than in short RF pulse, the spillover effect due to RF excitation is expected to decrease. Therefore longer readout RF acquisition would result in a narrower Z-spectrum. However, in single spin-echo simulation, a trend was found that longer RF pulse lengths would result in slightly broader Z-spectra. First possible reason is that the direct saturation due to single RF excitation is relatively minimal. The influence is too weak to make any observable change on the Z-spectrum. Second, longer RF pulse excitation also means the chemical exchange duration is prolonged. More exchangeable saturated protons, including MT pool and CEST agent, could exchange with bulk water during excitation, thus slightly reducing the signal ratio of Z-spectrum and broadening the Z-spectrum. In this manner, the spillover effect and chemical exchange process are two competitive factors. Although longer RF pulse introduces a smaller bandwidth, the chemical exchange during the pulse may overwhelm the contribution from direct spillover effect. Therefore, longer RF pulse is possible to give a slightly broader Z-spectrum, as reveal in simulation results. The variation found in MTR_asym_ may also due to these two factors.

In gel phantom study, however, the trend in computer simulation didn’t present. There was no statistical significant difference among Z-spectra while changing the readout RF pulse lengths. As mentioned previously, the noise level in phantom experiment is around 0.5%, where the 0.05% difference from simulation results would be impactless.

MTR_asym_ values didn’t show any apparent trend neither in computer simulation nor in phantom experiment. This indicates the Z-spectra broadening by long RF pulse length is a global impact on both positive and negative frequency offset regions. In other words, readout RF pulse generates similar signal changes on both sides of 0 ppm. Consequently MTR_asym_ will be insensitive to the RF pulse lengths since the effects of RF pulse are eliminated during calculation.

To examine if multiple refocusing RF pulse would enhance the impact of readout RF, both RARE simulation and phantom experiment were carried out. Simulation results demonstrate that the influence of refocusing number is minimal. Although 8 refocusing RF pulses readout gives a narrower Z-spectrum, the signal difference between 1 and 8 refocusing pulses at the edge of Z-spectrum is smaller than 0.0025%. To verify the impact of multiple refocusing RF pulses, a further computer simulation with 1 refocusing RF pulses and 128 pulses was carried out for comparison. The difference at the edge of Z-spectrum between 1 and 128 refocusing pulses is smaller than 0.0029%, which suggests that the multiple refocusing pulses still have a minor impact. Interestingly, phantom experiment shows that RARE acquisition would result in a slightly broader Z-spectrum, which is different to computer simulation results. As stated previously, the noise variation might cover the trend found in simulation. Both experiments indicate that refocusing RF pulse has a relatively minor impact on the Z-spectrum. Therefore the influence of refocusing RF pulse number can be regarded as a negligible factor.

Initially we hypothesized that the spillover effect might generate chemical exchange contrast during readout RF saturation, resulting in an additional chemical exchange effect and increasing the total CEST ratio. However, changing RF duration did not significantly alter Z-spectrum and MTR_asym_ in our phantom study. For *in vivo* imaging experiments, exchangeable molecules such as amides, amines, and other molecules will contribute to the CEST signal. The accumulative impact of readout RF in this complicated environment is speculated to be limited, since the signal difference in Z-spectrum is less than 0.05% in our 50 mM-creatine phantom study. The physiological fluctuation and measurement error of *in vivo* experiment could easily conceal this small difference. Therefore, it can be clearly identified that readout RF pulse length is too short to induce noticeable saturation transfer contrast, as stated in previous discussion^21^.

## Conclusion

In this study, the readout RF part of the CEST sequence was examined through both computer simulation and phantom experiment. We have demonstrated that readout RF pulses will not induce obvious saturation transfer contrast even with multiple pulses. Therefore, optimization of readout RF pulse length can be neglected in CEST imaging protocol.

## Methods

### Theory

A three-pool model referred to Desmond *et al.*’s work^22^ was used for computer simulation of CEST Z-spectrum. Seven Bloch equations were involved in describing the interactions between bulk water, CEST and MT pool. The signal contributions from CEST agent and MT pool are considered in the equations simultaneously to prevent inducing errors from a simplified linear assumption^23^. The Bloch-McConnell equations are shown below:


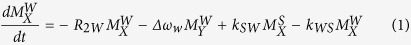



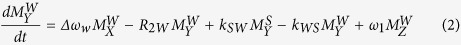



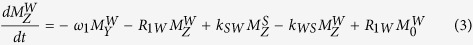



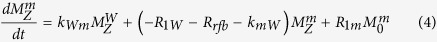



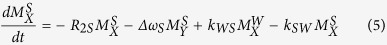



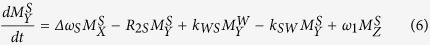



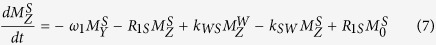


Where 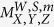 represent the Cartesian coordinate three dimension magnetizations of water (W), solute (S) and semi-solid MT pool (m), 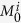 the equilibrium magnetization of each pool (i = W, S, m), the longitudinal relaxation rates, 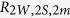 the transverse relaxation rates, 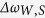 the offset frequency of the RF irradiation with the respect of water pool and solute pool, 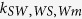 the chemical exchange rates between the three exchange pools, 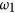 the power of saturation RF pulse (in Hz). 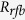 is the RF absorption profile for the MT pool and we have a Gaussian line shape expression according to Swanson’s work^24^ :


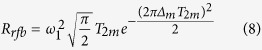


Where 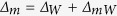, is the frequency offset for the asymmetric MT pool.

Among the Bloch equations, the transverse magnetization exchange in the MT pool was not considered since the T2 of MT pool is short enough to reduce the signal to a negligible value before the exchange is occurring. The effects of the remaining transverse magnetization terms were included in the 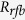 term.

### Simulation

All computer simulation experiments were performed on a personal computer. MATLAB function *ode45* (The MathWorks, Inc. Natick, MA. USA) was used for solving the seven ordinary differential equations (ODE) with the relative tolerance value of 10^−6^. The parameters of the three-pool model for 2 ppm Creatine signal were: 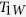 = 2.5 s, 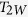 = 50 ms, 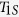 = 0.77 s, 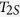 = 33 ms, 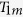 = 1 s, 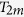 = 15μs, 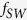 = 0.002, 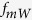 = 0.025, 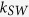 = 100 Hz, 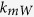 = 48 Hz, 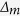 = −2.34 ppm, where 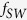 and 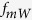 represents the magnetization ratio of solute to water and MT to water, respectively^17^,^20^,^22^. Six seconds continuous saturation irradiation with 1 μT was chosen to generate adequate CEST contrast. 101 points were simulated between −1000 and 1000 Hz with a step of 20 Hz. To simulate the spin-echo readout RF pulse, Gaussian pulse shape according to the shape file implemented on the scanner (Bruker Biospec 47/40, Ettlingen, Germany) was employed and the amplitudes of π/2 RF and π RF were calculated in each pulse duration. Five pulse lengths included 0.5, 1, 3, 5 and 10 milliseconds (ms) of both π/2 and π RF were simulated. TE was fixed at 62 ms. Moreover, in order to examine if multiple refocusing RF pulses would enhance the impact of imaging RF, simulation with 2, 4, and 8 π RF pulses were also performed to mimic sequences with multiple π RF pulses such as rapid acquisition with relaxation enhancement (RARE).

### Phantom Preparation

A dual 3% gel phantom with 50 mM creatine, and barbituric acid (Sigma-Aldrich Co. St. Louis, MO. USA) was prepared for this study. These two molecules, with chemical shifts of 2 and 5 ppm, respectively, relative to the water proton resonance at 0 ppm, were used to model a small chemical shift case. To prepare the phantom, the agarose solution was first heated to boiling and then cooled under room temperature till 70 °C. Then the contrast agent was added in and mixed properly. The pH value of each sample was adjusted by phosphate buffer. The pH value of creatine and barbituric acid solutions were respectively titrated to 6.98 and 7.10 monitored by an electronic pH-meter (SUNTEX Instruments, Taiwan). Proper pH value was used to achieve adequate CEST contrast. After titrating process was finished, these two samples were quickly transferred to two 6 mL glass test tubes each. The two glass tubes were then placed in a 50 mL centrifuge tube. The centrifuge tube was filled with pure 3% agarose solution in the outer compartment. The phantom was then left to solidify under room temperature before the experiment was conducted.

### MRI

**Figure 1:**
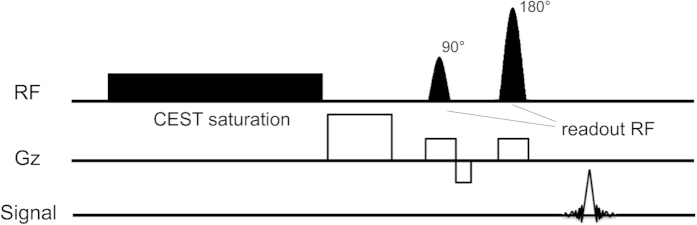
The basic CEST imaging scheme used in this study. A 4-second continuous wave saturation was performed followed by a spin-echo EPI acquisition.

**Figure 2:**
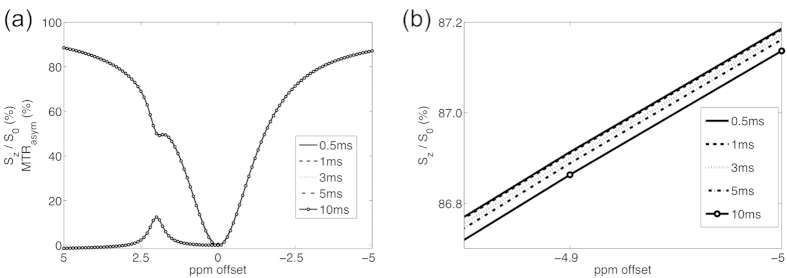
**(a)** Simulated Z-spectrum and MTR_asym_ through single spin-echo. No obvious difference was found among various RF pulse length. **(b)** The Z-spectrum between −4.85 and −5 ppm. Long RF pulse length would result in a slightly broader Z-spectrum. For example, signal difference of Z-spectrum between 0.5ms and 10ms is 0.05% at −5 ppm offset.

**Figure 3:**
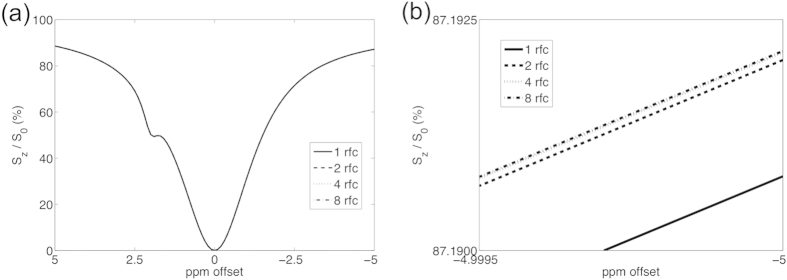
(a) Simulated Z-spectrum through multiple refocusing RF pulses. The Z-spectra are almost the same. (**b**) Enlarged figure reveals that more refocusing RF pulse results in a narrower Z-spectrum. Nevertheless, the difference is smaller than 0.0025%.

**Figure 4:**
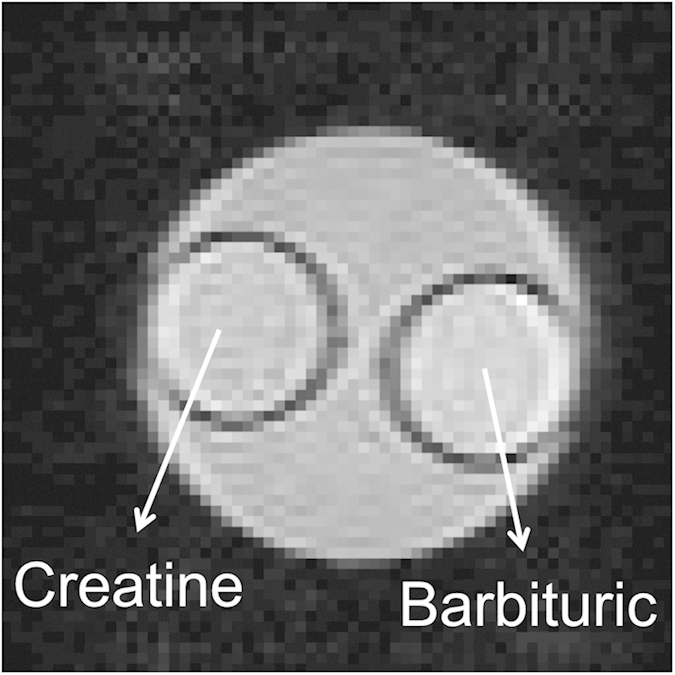
Phantom image acquired by EPI. This phantom includes three parts: creatine, barbituric acid and background gel.

**Figure 5:**
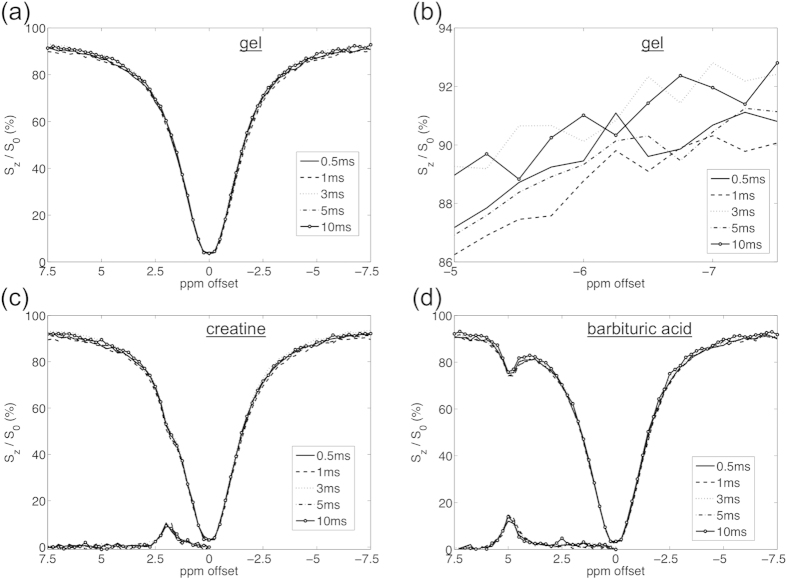
Phantom Z-spectra obtained with various RF pulse lengths. (**a,b**) shows the Z-spectra from background gel. No substantial difference was found among various RF lengths, while there was no obvious trend either. (**c,d**) shows the Z-spectra and MTR_asym_ of creatine and barbituric acid. In MTR_asym_ there is no significant difference among various RF pulse lengths.

**Figure 6:**
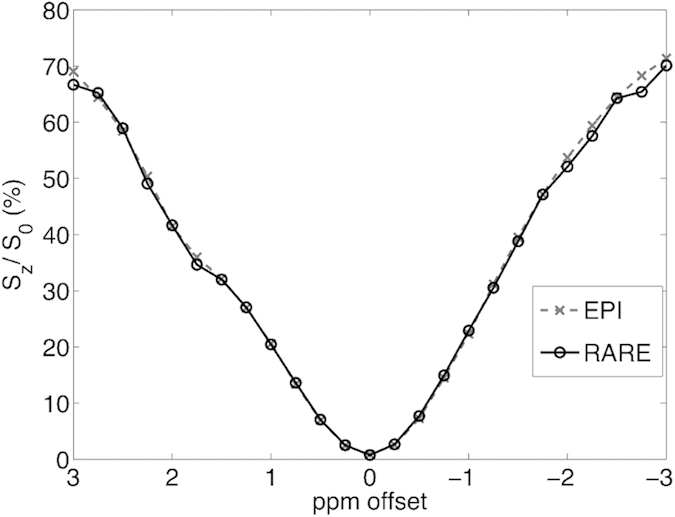
Z-spectra acquired by EPI and RARE were compared. The Z-spectrum obtained by EPI was slightly narrower than the Z-spectrum acquired by RARE.

All the experiment and images were carried out using a 4.7 T scanner (Bruker Biospec 47/40, Ettlingen, Germany). A volume RF coil was used for both transmitting and receiving. The central frequency and shimming parameters were automatically adjusted with the built-in program. The Fastmap shimming procedure was performed to enhance the field homogeneity. The imaging scheme includes a 4-second continuous wave irradiation pulse, which is followed by single-shot spin-echo echo-planar imaging (EPI) (as illustrated in Fig. 1). Both the excitation RF pulse and refocusing RF pulse in the EPI sequence were Gaussian pulses, with equal RF durations throughout the experiment. The various RF durations were 0.5, 1, 3, 5, and 10 ms. The corresponding bandwidth in full width of half maximum (FWHM) of the π/2 RF: 5480, 2740, 913, 548, and 274 Hz, respectively. (Table 1, refer to the user manual file of Bruker.) Imaging parameters in this experiment are as follows: repetition time (TR)/echo time (TE) = 11.5 s/45.83 ms, slice thickness = 2 mm, field of view (FOV) = 5 cm, matrix size = 64 × 64, saturation RF power = 1.25μT. The off-resonance frequencies of CW RF saturation were swept between ± 1500 Hz of water frequency with a step of 50 Hz. The reference S_0_ image in each Z-spectrum set was obtained by setting the offset frequency to 40000 Hz. For RARE experiment, the scanning parameters were all identical to EPI. The RARE factor was set to 8 and the effective TE (eTE) was 45.83 ms. All of the image data were processed on a PC with self-written MATLAB code. Region of interest (ROI) was selected and all the pixels in each ROI were collected for signal averaging or for statistical comparisons. Statistical analysis was made by comparing the signal at each chemical shift using student t-test.

## Additional Information

**How to cite this article**: Huang, S.-M. *et al.* Investigation of Readout RF Pulse Impact on the Chemical Exchange Saturation Transfer Spectrum. *Sci. Rep.* **5**, 15062; doi: 10.1038/srep15062 (2015).

## Figures and Tables

**Table 1:** Readout RF durations, the corresponding full width half maximum (FWHM) frequency bandwidths and impacts by EPI readout are listed below.

RF length (ms)	Bandwidth (Hz) 90°/ 180°	Simulated Z-spectrum value difference from 10 ms at −5 ppm offset	Phantom Z-spectrum value difference from 10 ms at −5 ppm offset	Simulated MTR_asym_ difference from 10 ms at 2ppm Creatine	Phantom MTR_asym_ difference from 10 ms at 2 ppm Creatine
0.5	5480/3220	0.05%	−1.79%	0.01%	1.28%
1	2740/1610	0.04%	−2.72%	−0.03%	0.07%
3	913.3/536.7	0.03%	0.30%	−0.07%	0.38%
5	548/322	0.02%	−2.05%	0.01%	0.10%
10	274/161	–	–	–	–

In simulation results, a trend was found and the difference of Z-spectrum value between 0.5 ms and 10 ms RF is 0.05% at −5 ppm offset. In phantom results, no specific trend was found along with RF durations since the trend found in simulation results may be covered by noise. As to the MTR_asym_ of creatine, no specific trend was found neither in simulation nor phantom experiment.
